# Read my lips! Perception of speech in noise by preschool children with autism and the impact of watching the speaker’s face

**DOI:** 10.1186/s11689-020-09348-9

**Published:** 2021-01-05

**Authors:** Rochelle S. Newman, Laura A. Kirby, Katie Von Holzen, Elizabeth Redcay

**Affiliations:** Department of Hearing and Speech Sciences, University of Maryland, 100 Lefrak Hall, College Park, MD 20742 USA

**Keywords:** Autism, Noise, Speech perception, Face

## Abstract

**Background:**

Adults and adolescents with autism spectrum disorders show greater difficulties comprehending speech in the presence of noise. Moreover, while neurotypical adults use visual cues on the mouth to help them understand speech in background noise, differences in attention to human faces in autism may affect use of these visual cues. No work has yet examined these skills in toddlers with ASD, despite the fact that they are frequently faced with noisy, multitalker environments.

**Methods:**

Children aged 2-5 years, both with and without autism spectrum disorder (ASD), saw pairs of images in a preferential looking study and were instructed to look at one of the two objects. Sentences were presented in the presence of quiet or another background talker (noise). On half of the trials, the face of the target person speaking was presented, while half had no face present. Growth-curve modeling was used to examine the time course of children’s looking to the appropriate vs. opposite image.

**Results:**

Noise impaired performance for both children with ASD and their age- and language-matched peers. When there was no face present on the screen, the effect of noise was generally similar across groups with and without ASD. But when the face was present, the noise had a more detrimental effect on children with ASD than their language-matched peers, suggesting neurotypical children were better able to use visual cues on the speaker’s face to aid performance. Moreover, those children with ASD who attended more to the speaker’s face showed better listening performance in the presence of noise.

**Conclusions:**

Young children both with and without ASD show poorer performance comprehending speech in the presence of another talker than in quiet. However, results suggest that neurotypical children may be better able to make use of face cues to partially counteract the effects of noise. Children with ASD varied in their use of face cues, but those children who spent more time attending to the face of the target speaker appeared less disadvantaged by the presence of background noise, indicating a potential path for future interventions.

## Background

Autism spectrum disorders (ASD) are a diverse set of conditions that often include reduced and atypical receptive language abilities [1]. Children diagnosed with or at risk for ASD may approach the task of learning words differently than do their typically developing peers (see [2, 3] for recent reviews) and are less likely to look at an object upon hearing its name [4–6]. They also access semantic meaning later in the word recognition process [7]. Typically developing children show a gradual improvement in the speed of their word recognition over the first years of life [11, 12], but children with ASD show no corresponding gains, regardless of age or symptom severity [8]. Individuals with ASD show abnormalities in sound and speech-sound processing [9, 10, 13–15], which may lead to such lexical delays [2, 16, 17]. For example, adolescents with ASD have been reported to show difficulties (1) discriminating high-frequency sounds [18], a critical skill for distinguishing fricatives such as /s/ and /∫/; (2) detecting silent gaps [18], which are crucial for identifying stop consonants; (3) attending to one sentence in the midst of distraction [19], which would likely impact their speech recognition in many real-world situations (such as classroom settings, where noise is a common occurrence [20, 21]). Their phonetic categorization appears to be less specialized for their native language [19]. Finally, individuals with ASD failed to show a typical electrophysiological response to vowel changes [13] or syllable changes [22], suggesting that differences between speech sounds were less salient for them. All of these differences point to a pattern where individuals with ASD would be less likely to detect differences between speech sounds, particularly in difficult listening conditions (see [14] for a review).

In the current paper, we focus on a real-world situation that poses a challenge to any listener: understanding speech in the midst of background noise. Modern households and classrooms include many sources of potential noise, and the ability to separate speech from background noise and attend selectively to the former is a critical skill for understanding spoken language in such environments [23]. Adolescents and adults with ASD appear to have particular difficulty recognizing speech in noisy environments (e.g., [19, 24]), although some evidence suggests that children with ASD have general deficits in speech recognition, regardless of the presence or absence of noise [25, 26]. The ability to understand speech in noise is perhaps even more important for young children, who are still actively learning their native language, yet little research has investigated this [27].

This difficulty with listening in noise appears particularly problematic when the noise itself varies over time [24], such as is the case for human speech (compared to, say, the background noise produced by a refrigerator humming). In situations of time-varying background noise, neurotypical adults focus their attention on the portions of the signal where the noise is softest, referred to as “dip listening,” and then integrate those fragments across time, whereas adolescents with ASD struggle in such situations [28]. Children with ASD show less efficient segregation of sound streams than do age-matched peers [27] and fail to use acoustic cues to segregate the two sound sources, contrary to age-matched peers [29]. Individuals with ASD have a specific difficulty listening to and segregating the speech of one talker when another person speaks at the same time.

Difficulty in understanding speech in the presence of background noise could lead to difficulties in learning language, at least for children who are frequently in noisy environments. The inability to focus attention selectively would theoretically result in fewer effective opportunities to learn, as much of the language input that young children receive occurs in the presence of background noise, both in public settings [30] and in school classrooms [23, 31, 32]. The ability to separate speech from background noise is thus a critical skill for understanding spoken language in acoustically complex environments, and children already at elevated risk for language deficits (such as those with ASD) may be particularly vulnerable. In addition, many people with ASD show reduced or atypical executive function abilities [33, 34], including difficulties with attention, a skill necessary for comprehending speech in a noisy environment. Some researchers have suggested that the auditory and/or speech differences in ASD are specifically the result of a difficulty in focusing attention on a particular sound while filtering out other, competing sounds [10, 35]. Thus, there are many reasons to suspect that background noise may be especially problematic for young children with ASD.

One of the cues that typically developing infants and adults use to help them separate streams of speech is visual information from the talker’s face [36, 37]. Listeners generally perform better in noisy environments when they can see the face of the speaker [37]. Whether this would be the case for children with ASD is less clear. Several studies have suggested that individuals with ASD show atypical gaze to faces ([38], see [39] for a review), including to the faces of people talking [40]. For example, in the latter study, children with autism showed a different distribution of eye gazes to people talking than did neurotypical children, one generally associated with younger children and a less stable (or less fully self-organized) processing system. However, some of the other reported atypicalities involve a greater focus on a speaker’s mouth than on his/her eyes [41, 42] (but see [43]), which might actually be helpful when listening to speech in noise, and is seen in neurotypical children during key developmental phases of language acquisition [44]. Yet, in a sample of adolescents with ASD, presentation of the speaker’s face did not facilitate perception of speech in noise to the same degree that it did with their neurotypical peers [45]. This might suggest that children with ASD would have similar difficulties, which could, in turn, exacerbate any difficulties listening to speech in the presence of distractors^1^. Indeed, children with autism are less influenced by visual information when viewing audio-visual speech with conflicting auditory and visual information [46–48], The reduced use of visual information may be the result of reduced use of lip reading in this population, which would presumably also impact the use of visual facial information when listening in noise, although studies have found varying results in this regard [45, 47, 48]. Moreover, Irwin and Brancazio [75] found that individuals with ASD, aged 6-16 years, spent less time watching the face, and in particular the mouth, of a speaker in a listening-in-noise task than did a typically developing age- and language-matched control group, likewise suggesting that the presence of visual information would not help them.

The current study compared children with ASD with age- and language-matched peers on their recognition of familiar words in the presence of background noise (here, the presence of multiple people speaking) when the speaker’s face was present and absent. We focus on children aged 2-5 years of age, an age range typically showing great vocabulary growth and where difficulty processing spoken language is likely to have far-reaching implications for subsequent language development. Age-matched peers were included as children with ASD are more commonly compared with this group in the classroom setting. We include language-matched peers to account for anticipated differences in vocabulary skills compared to age-matched peers, which could potentially impact performance in a lexical task. Finally, we use a looking-based paradigm as our method of testing; this paradigm does not require children to make explicit identification judgments and is particularly suited to capture comprehension in individuals who may have difficulty with expressive language and tasks involving explicit responses [8, 49]. In addition to a more traditional analysis of overall accuracy, we analyzed differences in children’s dynamic-looking patterns over the course of the entire trial using growth curve analyses. Considering children with ASD show a different pattern of access to semantic representations during word recognition than neurotypical children [50], we used growth curve analyses, in addition to overall accuracy measurements, to capture children’s dynamic-looking patterns over the course of the entire trial. The latter is especially useful to assess whether children’s identification of the correct object was slowed when the label was presented in noise.

We had three predictions (1) Children with ASD would show overall poorer accuracy than their peers at identifying known words when presented in a multi-talker environment compared to quiet listening conditions. (2) When the target speaker’s face was present, children with ASD would show a smaller benefit in word recognition compared to neurotypical children. (3) This difference would be even greater when speech was presented in background noise, with neurotypical children showing stronger performance in noise when a face was present than children with ASD.

This work serves to investigate whether children with ASD have greater difficulties listening in noise than do their peers. If so, this could have profound implications for classroom learning and could suggest the need for classroom modifications.

## Methods

### Participants

Three groups of children participated in this study: (1) seventeen children who met criteria for autism spectrum on the Autism Diagnostic Observation Schedule (ADOS-2) [51] conducted by a research-reliable administrator who was highly trained and experienced in assessing ASD, and had been previously diagnosed clinically and had no known comorbidities; (2) seventeen typically developing children matched for chronological age (CA), and (3) thirteen typically developing children matched for language age (LA) (see below for more information). General demographic information is in Table 1.

**Table 1:** Demographic properties of participant groups

Group	Age when tested	Gender	Mullen Receptive Language raw scores	Primary caregiver years education*	Racial/ethnic background*
Autism (*n* = 17)	50.3 m (10.6)	11 m, 6 f	28.6 (10.3)	16.7	60% Caucasian, 33% Black, 7% Asian; 7% Hispanic
Age-matched controls (*n* = 17)	50.1 m (10.5)	11 m, 6 f	42.0 (6.1)	17.0	50% Caucasian, 13% Black, 31% Asian, 6% American Indian; 13% Hispanic
Language-matched controls (*n* = 13)	35.5 m (12.8)	7 m, 6 f	32.9 (7.3)	17.8	73% Caucasian, 18% Black, 9% Asian; 18% Hispanic
Autism subset matched for language (*n* = 13)	51.5 m (9.9)	9 m, 4 f	32.5 (7.3)	16.2	64% Caucasian, 27% Black, 9% Asian; 0% Hispanic

Numbers in parenthesis are standard deviations

*2 individuals with autism and 3 control participants did not complete questionnaires. Numbers do not total 100% because ethnicity and racial background were separate questions

One child with ASD was bilingual according to parental report between Afan Oromo (75% usage)^2^ and English (25%), and another was bilingual but majority English (70%)-French (30%); two control participants were similarly bilingual but majority English (English 70%/Tagalog 30% and English 80%/Spanish 20%); all other children heard at least 90% English in the home (a common definition for effectively monolingual). We opted to include these two bilingual children because we felt that lexical identification, with the simple words chosen here, would still be within their linguistic competence, and because matching for language skill should account for variability across groups. Informed consent was obtained from the parents of all participants in this study.

The children with ASD (11 male, 6 female) ranged in age from 29 to 64 months (or roughly 2 1/2-5 1/3 years). In addition to the ADOS, we also asked parents of children with ASD to complete the Child Behavior Checklist (CBCL1.5-5 [52]) and the Social Communication Questionnaire (SCQ [53]). Detailed information about the children with ASD is shown in Table 2. Children in the CA group (11 male, 6 female) were individually matched to be within 2 months by age; there was no difference in average age between the two groups (*t* = 0.61, *p* = 0.55).

**Table 2:** Test results and specific demographics from children with ASD

Age when tested (in months)	Gender	ADOS Social Affect Score	ADOS Restricted and Repetitive Behaviors (RRB) score	Mullen Receptive Language raw scores	Social Communication Questionnaire	Child Behavior Checklist (CBCL) T score
29	M	6	3	12	14	61
31	M	10	1	21	13	63
37	F	11	0	29	23	63
44	M	17	4	11	Inc	41
44	M	15	4	28	16	64
46	M	14	1	26	Inc	Inc
49	M	13	16	34	18	65
51	M	14	4	41	25	65
52	F	6	3	30	Inc	56
54	M	6	4	42	13	54
54	F	5	2	42	12	69
59	F	8	2	41	13	59
59	F	18	6	26	18	61
60	F	12	2	12	17	71
61	M	18	4	26	23	72
61	M	14	7	30	13	57
64	M	19	3	36	11	52

*Inc* incomplete; some parents of participants were not able or unwilling to complete the ancillary measures

In order to match children by language skills, all children completed the receptive language measure of the Mullen Scales of Early Learning [54]. One child with ASD was unable to complete this assessment for scheduling reasons; three additional children with ASD had extremely low language ages (< 12 months) such that they could not be matched with another child without that child being too young to participate in the study. Thus, while these children were included in the analyses comparing children with ASD to age-matched peers, they were not included in the analyses comparing children with ASD to language-matched peers. This dual-analysis approach allows us to include data from children with low language abilities (a group that is often excluded from research), while still comparing children with ASD to those with similar language abilities. In cases where the two analyses give the same results, we can be assured that the findings are not driven by language differences between groups or by having a restricted diversity in our participants with ASD. This dual approach, however, means that the language-matched analyses are lower in power; they include the remaining 13 children with ASD and 13 typically developing children matched for Mullen receptive language scores (within 2 points). On average, Mullen scores were 32.5 for children with ASD and 32.9 for language-matched children without ASD, *t* = 1.44, *p* = 0.18. Unsurprisingly, the children with ASD tended to have lower Mullen scores than their age-matched peers (scores of 29 vs. 42; *t* (16) = 5.38, *p* < 0.0001) and tended to be older than their language-matched peers (51.5 vs. 35.5 months, *t* (12) = 4.19, *p* < 0.005).

Five typically developing children were included in both matched groups—as age-matched controls for one child, but as language-matched controls for a different child. Because we conducted these as entirely separate comparisons, the overlap across sets does not reduce statistical variability and thus was not deemed to be a concern. As a result, data from a total of 41 children (17 with ASD, 12 who were only age-matches, 8 who were only language-matches, and 5 who were both) are actually included in this paper. In order to find appropriate language-matched children, a larger number of typically developing children were tested than were actually included. Of the children originally recruited, data from four children with ASD were excluded when the children’s ADOS scores did not meet the criteria for autism spectrum on the ADOS (despite having received a prior diagnosis). Finding such children is not unexpected, since many of these children had been in intensive clinical therapy for several years; successful therapy can result in children no longer matching diagnostic criteria, even when ongoing support remains necessary. Three more children with ASD were excluded because they could not be scheduled for ADOS testing, although they, too, had a prior diagnosis. In order to be absolutely sure that our participants with ASD met current research diagnostic criteria at the time of testing, we opted to exclude these children from analyses. Another participant who was intended as a control participant was excluded because of extremely low Mullen language scores and a suspicion of autism. Finally, data were lost as a result of equipment or experimenter errors for 4 children who were potential control participants.

### Stimuli

All words used in the study were selected on the basis of being typically produced by at least 75% of children by age 24 months according to MCDI norms [55, 56]; this ensured that they would be likely to be well-understood by our participants. Visual stimuli consisted of digital images of these words, presented in pairs. Image pairs were matched for approximate size and color to avoid saliency differences. There were 6 possible object pairs on the test trials: *baby* paired with *doggie*, *book*—*fish*, *flower*—*apple*, *shoe*—*car*, *sock*—*cup*, and *truck*—*ball*. An additional two object pairs were used for practice trials: *horse*—*bottle*, and *tree*—*chair*.

Given that one of our goals was to compare comprehension in quiet with that in noise, we presented a target speaker naming these objects which was sometimes blended with a distractor voice speaking at the same time (the noise). The target stream consisted of a single female talker producing 2 sentences: “Look at the ___! Where’s the ___?” The distractor consisted of a single female voice reading a 1-sentence passage. A single voice was chosen because this form of noise was found to be particularly problematic in prior studies with adolescents and adults with ASD [24]. The distractor passage came on at the same time as the target speech stream and faded out after the completion of the speech passage. Sentences were recorded in a noise-reducing soundbooth (Shure SM81 microphone, 44.1 kHz sampling rate, 16 bits precision), adjusted to be the same duration and RMS amplitude, and were combined at a 5 dB SNR. This noise level is akin to that found during book-reading time across five occupied toddler classrooms ([69]; personal communication), making it a realistic, if possibly idealized^3^, representation of typical language-learning situations. This level has also been reported as being a common one for speech in many noisy environments [57]. The target word occurred 1500 ms subsequent to the start of the trial; the total trial duration was 8 s.

Our second goal was to examine whether children benefitted from being able to see the speaker while she was producing the target stream. We therefore video recorded the target speaker while producing the sentences above and presented this video on half of the test trials. It appeared at the start of the trial and was located in the center of the screen, between the two objects. The face disappeared at the 6-s mark, after completion of the speech; thus, the final 2 s of the trial contained only the two object choices.

### Experimental procedure

To understand how the recognition of words by children with and without ASD is impacted by background noise as well as whether this is modulated by a video of the speaker producing the target sentence, we used an adaptation of the Intermodal Preferential Looking Procedure [58]. This procedure has been used for decades to study lexical comprehension in children [59] and variants have recently been used in a variety of studies with children with ASD [4, 8, 60]. Children sat on their caregiver’s lap, facing a widescreen TV. At the start of each trial, an attention getter (screen saver plus classical music) appeared to attract the participant’s attention. Subsequently, children saw a pair of images, on the left and right sides of a large-screen video monitor at ~20 degrees visual angle. At the same time, they heard an auditory stimulus at approximately 65-70 dBA (all items were matched for RMS amplitude prior to presentation) instructing them to look at one image in particular; we expect that they will spend more time looking at the correct image than the foil image if they recognize the target words.

The first two trials were practice trials and did not include a distractor (background) sound. On one of the trials, the child saw a face appear in the center of the screen to present the target sentence, while on the other trial they simply heard the target sentence and did not see the speaker.

This was followed by 24 test trials. Test trials could occur either with distractor speech in the background, or without, and either with the face present or without, for a total of 4 conditions (6 trials each). These are henceforth referred to as the Face-Quiet, Face-Noise, NoFace-Quiet, and NoFace-Noise conditions. Each of the 6 object pairs occurred one time in each of the four conditions. Across those four tokens, each object within the pair served as the correct answer twice, and each appeared on the left side (vs. the right side) of the screen twice. Trial order was randomized for each individual. We examined what percentage of time children looked at the appropriate (vs. inappropriate) object as a measure of his or her ability to understand the speech, with a looking percentage to the target above 50% indicating recognition. The caregiver listened to masking music over headphones throughout the study to prevent any biasing of the child’s behavior.

### Coding

Two coders, blind to condition^4^, individually coded each child’s looking behaviors on a frame-by-frame basis using the Supercoder coding software [61]. The first 1500 ms (45 frames) of each trial occurred prior to the first presentation of the target word, to allow children the opportunity to view each object before naming. Since children could not know which object to look at until hearing the word, these initial 45 frames were excluded from analyses of target looking. On trials without a face, children had two items they could look at (the two digital images); on trials with a face, they could potentially look at three objects (left and right images and center face). Coders were blind to which side was correct, and to trial type (noise vs. not), but were not blind to whether there was a face on the screen (i.e., a potential third target) or not.

If the two coders disagreed on any trial by more than 20 frames (2/3 s), a third coder was engaged. The averages of the two closest codings were used as the final data. This occurred on 48 of the trials for the 17 children with ASD (or on 11.76% of trials), 21 trials for the 13 language-matched children (6.7%), and 38 trials (9.3%) for the age-matched children. If a child did not look to either of the two objects on a trial (such that a proportion of the correct looking would require division by zero) or did not look at them for a minimum of 10 frames combined (1/3 of a second), that trial was excluded from the overall data. This occurred on a total of 14 trials across the participants with ASD, 13 trials across the age-match controls, and 9 trials across the language-match controls.

We first checked to make sure children with ASD did not have an extreme side bias that might impact their task performance. Looking across all trials, all of the children looked at least 35% of the time to each of the two sides, suggesting they were willing to look to both sides of the screen.

We analyzed the data in two separate ways. In the first, we looked at simple accuracy, defined as the proportion of time that infants remained fixated on the picture of the target object, rather than the foil object, subsequent to the onset of the target word. Although some studies with typically developing children have used limited time windows for a simple accuracy analysis, we were concerned both that children with ASD might have a different (or slower) processing window, and that noise might slow children’s processing. Furthermore, children might have different time scales for trials with vs. without a face present. For these reasons, we felt that a short analysis window was making assumptions that might not be valid; we therefore conducted analyses using the entirety of the trial as the time window.

The advantage of this accuracy approach is that it better matches prior studies with typically developing children. However, we also conducted a second; more in-depth analysis of the time-course of children’s looking, based on growth-curve modeling. Unlike the accuracy approach, growth-curve modeling allows for an examination of target looks between conditions and groups as they unfold over the course of the trial. Target fixation proportion (as coded by a single coder) was logit transformed using an adjustment for any data that was exactly 0 or 1. Although simple accuracy is typically reported in the Intermodal Preferential Looking Paradigm [58], when considering target looks at each time point confidence intervals around estimates of proportion values can fall outside of physically possible values (less than 0, more than 1), whereas adjusted logit transformations (henceforth target fixations or looks) take this into consideration (for further explanation, see the Windows Analysis Vignette in the eyetrackingR package [62]). For ease of interpretation, looking estimates above 0 indicate looks to the target (or face), while looks below 0 indicate looks to the incorrect image (or away from the face).

We examined toddlers’ target looks in the post-naming phase (after onset of the target word at 1500 ms) using growth curve analysis (GCA). Mirman and colleagues [63, 64] describe the application of GCA to eye-tracking data analysis. It has also been applied to describe target looks in toddler word recognition [65–67]. In contrast to mean proportions of looks after onset of the target word, GCA allows us to describe the shape of change in target looks over time, which can more finely reflect the cognitive processes involved in word recognition. We examined target looks from the onset of the target word (1500 ms into the trial) to an end point of 2000 ms after target word onset (3500 ms into the trial). Although previous studies have used a 1500 ms time window [65, 68], we used a longer 2000 ms time window to ensure we captured variation introduced by non-typical language processing (i.e., children with ASD) and the large age range tested.

We use GCA to capture the change in target looks over time as a function of a set of predictors. To create the growth curve model, the changes in target looks over time were submitted to a mixed effects model. Visual inspection of the raw data revealed multiple bends in the time course of responses, suggesting at least a cubic shape. As a result, we used first, second, and third-order orthogonal polynomials (linear, quadratic, cubic) to estimate the steepness (linear), sharpness of the peak (quadratic), and sharpness of the two peaks (cubic) across responses ([63] pp. 49-50).

A series of models were computed. In all models, the fixed effects included condition (noise, quiet) and group (typical, ASD). All fixed effects were dummy coded. Across all models, “Quiet” and “Typical” were coded as the reference conditions. Participant and participant-by-noise random effects were included on linear and quadratic polynomial-time terms, unless otherwise noted.

## Results

The following sections present the results of the overall accuracy and growth curve analyses. Unless comparisons with language-matched peers showed a different pattern of results, analyses are reported for age-matched peers and children with ASD.

### General task performance

To ensure that children understood the task and the target words, we first examined their performance in quiet, without a face (Face-Quiet; the simplest condition). As expected, both children with ASD and their age-matched controls showed above-chance performance for these well-known words, looking longer to the correct object than the incorrect object (children with ASD looked to the correct object 66.3% of time, *t* = 4.79 *p* < 0.001 when compared to a 50% chance criterion; age-matched controls 68.1% of time, *t* = 5.47, *p* < 0.001; these values are in the typical range for this task (see [58]).

### Listening in noise, face absent

Our primary questions had to do with performance in the noise conditions: If children with ASD are particularly hampered by background noise, we would expect them to show poorer looking than their peers in this condition. When the face was absent, there was no significant difference between conditions (*F* (1, 32) = 1.71, *p* = 0.20), although accuracy was poorer in noise (63.9%) than in quiet (67.2%). Interestingly, this effect was significant when children with ASD were compared with their language-matched peers (*F* (1, 24) = 4.31, *p* < 0.05; 63.5% vs. 68.5%), suggesting that the impact of noise is less apparent when some children have high language skills. The lack of a group×condition interaction, however, suggests that the effect of noise is common to both children with and without ASD.

**Fig. 1:**
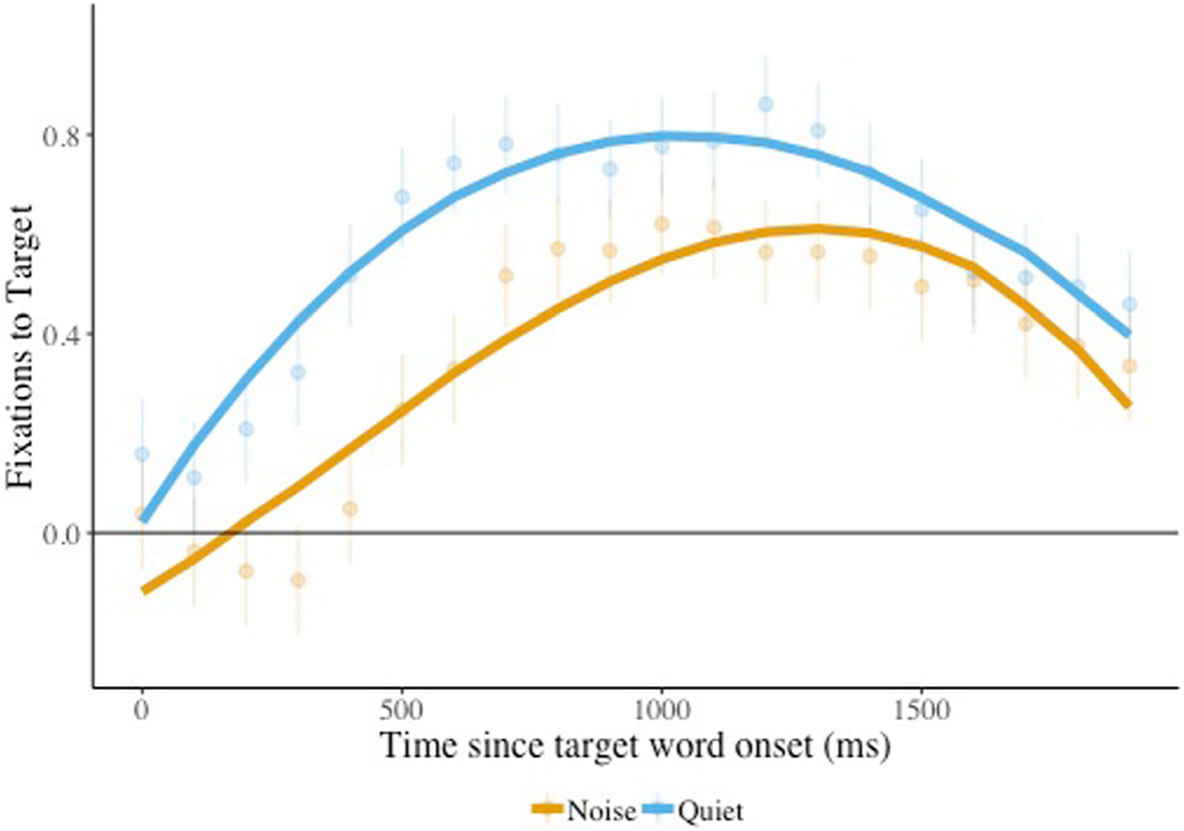
Target looks, effect of condition, face absent. Target image fixation is plotted for listening in noise vs. quiet (condition) when the face was absent, combining children with ASD and age-matched peers. The solid lines indicate the model fits for the significant effects including condition. Raw means and standard errors are plotted underneath the model fits. Estimates above 0 indicate looks to the target

If children with ASD are particularly impaired by noise, they should be slower than their peers to look appropriately when noise is present—which could be captured in a growth curve analysis. The resulting model (Appendix 1) revealed a significant effect of condition (*β* = −0.23, *SE* = 0.11, *p* = 0.05). As can be seen in Fig. 1, looks to the target were greater for trials presented in quiet compared to noise. The presence of noise reduced word recognition, but this did not differ between children with ASD and their age-matched counterparts.

### Listening in noise, face present

We next examined whether the presence of a face helps to counteract difficulties listening in noise. In the overall accuracy analysis, when the face was present there was a marginal effect of group (*F* (1, 32) = 3.18, *p* = 0.084), with poorer performance by children with ASD (59.8% vs. 68.2%).

**Fig. 2:**
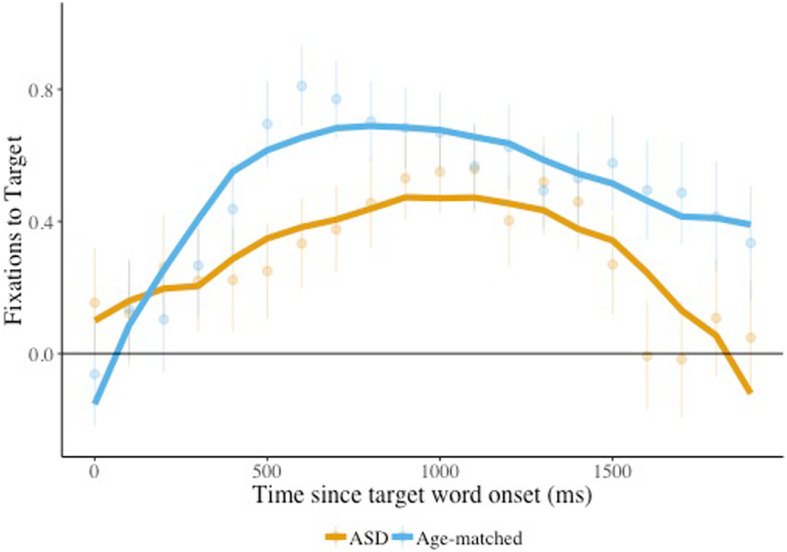
Target looks, effect of group, face present. Target image fixation is plotted for children with ASD and age-matched peers (group) when the face was present, combining listening in quiet and noise conditions. The solid lines indicate the model fits for the significant effects including group. Raw means and standard errors are plotted underneath the model fits. Estimates above 0 indicate looks to the target

The results of the growth curve model (Appendix 2) revealed a significant interaction between group and the cubic time term (*β* = −0.52, *SE* = 0.26, *p* < 0.05). As can be seen in Fig. 2, the peak for age-matched children was much sharper than that for children with ASD, suggesting faster acceleration in looks to the target for the former group. This might be an indication that children with ASD were simply less quick to look away from the face and toward the target object (that is, that they remained fixated on the face for longer).

**Fig. 3:**
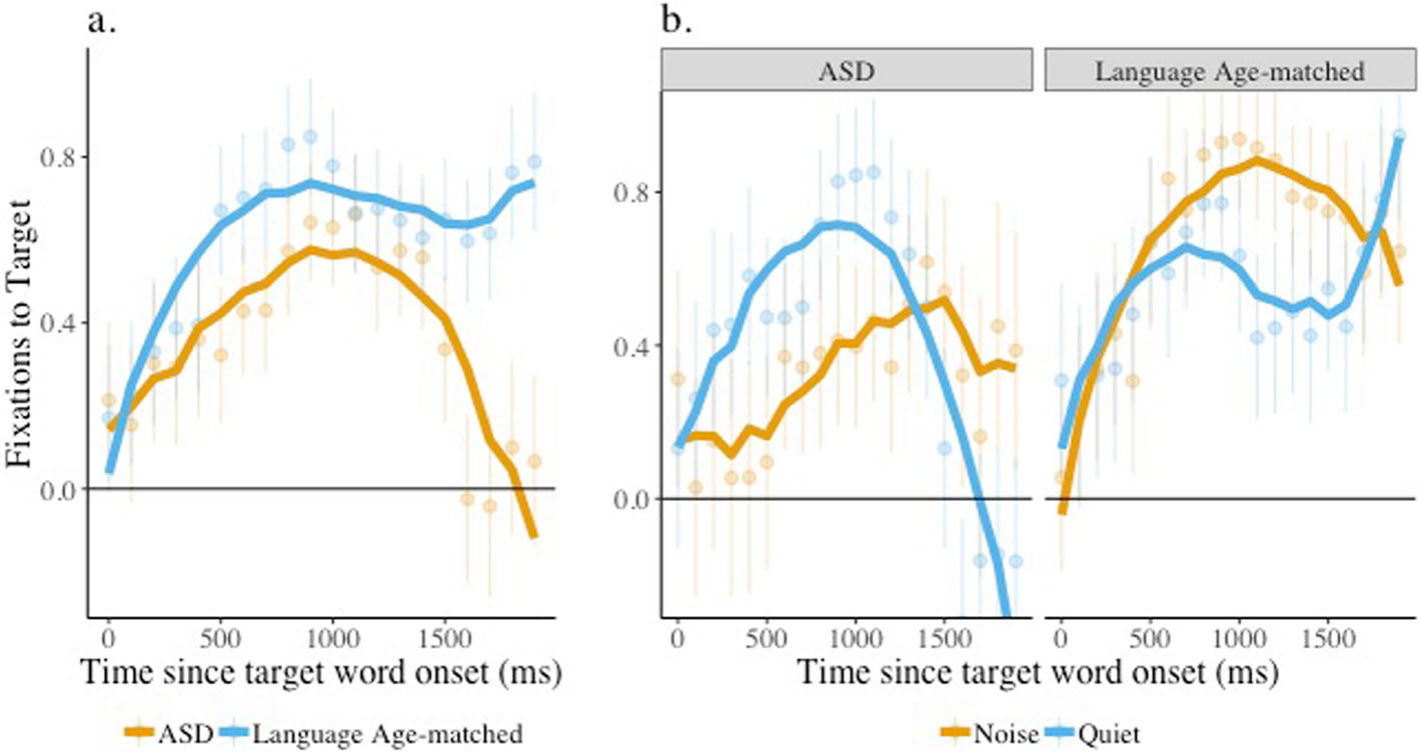
Target looks, effect of condition and condition by group interaction, face present. Target image fixation is plotted from children with ASD and language-matched peers (group) listening in noise vs. quiet (condition) when the face was present. The solid lines indicate the model fits for the significant effects including group (**a**) and the interaction between group and condition (**b**). Raw means and standard errors are plotted underneath the model fits. Estimates above 0 indicate looks to the target

The results of a similar model comparing language-matched children and children with ASD revealed a significant interaction between group and both the quadratic (*β* = −1.26, SE = 0.54, *p* = 0.02) and cubic (*β* = −0.69, SE = 0.31, *p* = 0.03) time terms. Similar to the age-matched comparison, the peak for language-matched children was sharper than that of the children with ASD (Fig. 3a), suggesting faster acceleration in looks to the target for the former group. Yet, in this analysis, the interaction between noise, group, and the quadratic time term was also significant (*β* = 1.83, SE = 0.73, *p* = 0.02). As can been seen in Fig. 3b, the peak of target looks on trials presented in noise was higher for language-matched children compared to children with ASD, although the two groups showed little difference in performance on trials presented in quiet. These results suggest that noise impacted target looks to a greater extent in children with ASD than their language-matched peers.

### Interim summary

Based on the results of the previous sections, we see that noise impaired the performance of both children with and without ASD; children looked less accurately, and looked to the appropriate object more slowly, when noise was present. When the face was absent, the effect of noise was generally similar across children with and without ASD. But when the face was present, the noise had a more detrimental effect on children with ASD than their language-matched peers. This could be an indication that neurotypical children are better able to make use of face cues to partially counteract the effects of noise; alternatively, it might be an indication that the face itself is attracting the attention of children with ASD, such that they look toward the appropriate object more slowly. The next section attempts to address this possibility.

### Attention to the face

To examine overall accuracy, we coded the number of frames that the child spent looking to the center of the screen (where the face was located) on trials for which faces occurred. Children with ASD did not spend any less time looking at the face than did their age-matched controls (no effects of condition, group, or their interaction, all *F* < 1). In the quiet condition, there was no difference in the time spent looking at the face between children with ASD (18.8 s) and their age-matched peers (19.2 s; *t* (16) = 0.12, *p* = 0.90). The noise condition showed a similar lack of a difference between children with ASD (19.3 s) and their age-matched peers (18.5 s; *t* (16) = 0.38, *p* = 0.71). This corresponds with the literature suggesting that while individuals with ASD have different gaze patterns than their neurotypical peers, these do not necessarily involve a failure to attend to faces [39, 41].

**Fig. 4:**
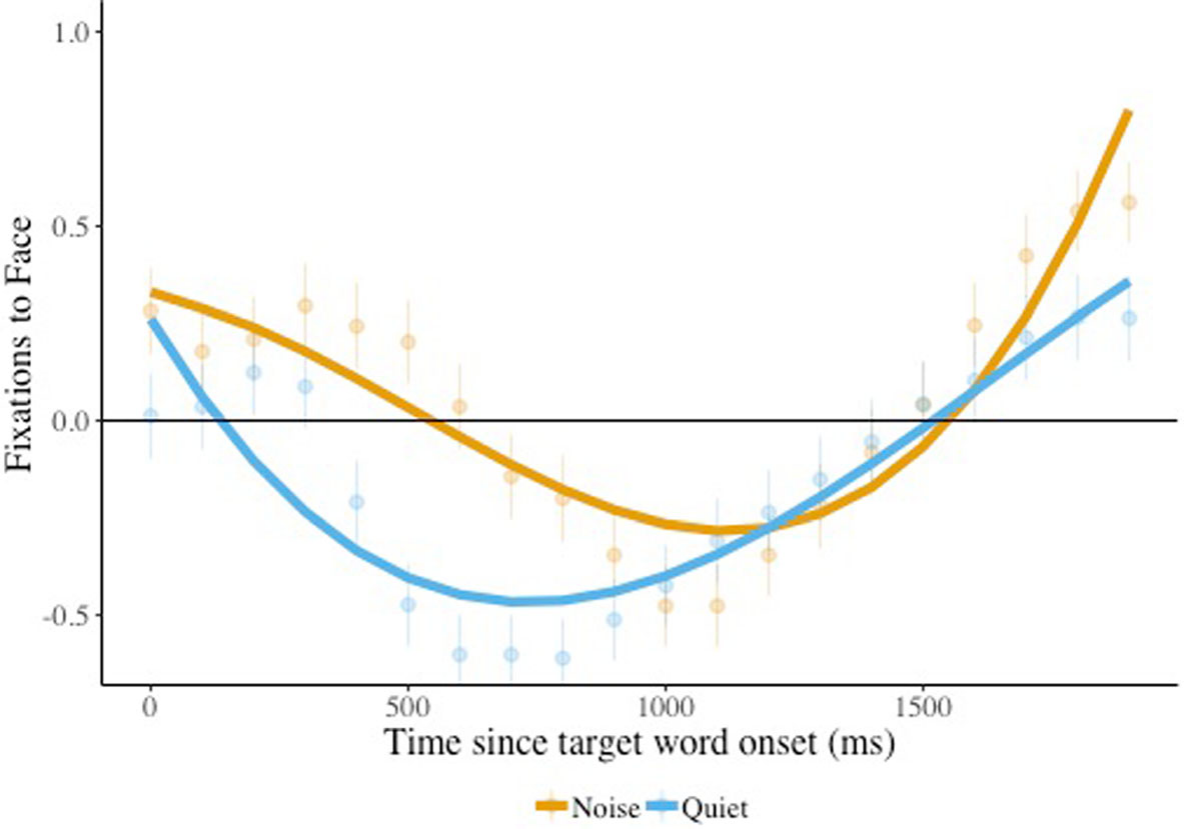
Face looks, effect of condition. Children’s looking to the speaker face is plotted, comparing listening in noise vs. quiet (condition), combining children with ASD and age-matched peers. The solid lines indicate the model fits for the significant effects including condition. Raw means and standard errors are plotted underneath the model fits. Estimates above 0 indicate looks to the target

We next used growth-curve analysis to examine children’s looks to the face over the whole time course. The results of this model (Appendix 3) revealed a significant interaction between condition and the cubic time term (*β* = 0.73, SE = 0.20, *p* < 0.0001). As can be seen in Fig. 4, the peak for trials presented in quiet was sharper than for trials presented in noise, suggesting faster acceleration in looks away from the face when the trial was presented in quiet, but that children remained looking at the face longer when the trial was presented in noise.

**Fig. 5:**
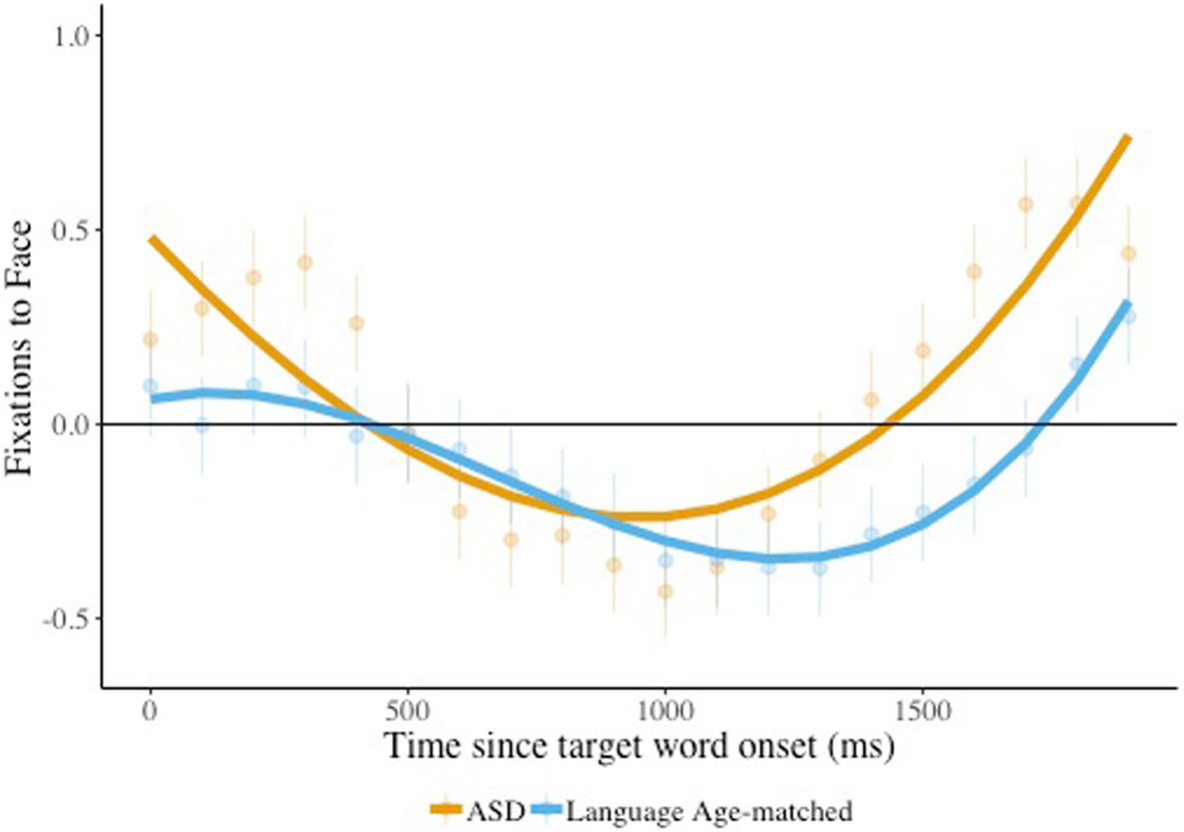
Face looks, effect of group. Children’s looking to the speaker face, comparing children with ASD and language-matched peers (group), combining listening in quiet and noise conditions. The solid lines indicate the model fits for the significant effects including group. Raw means and standard errors are plotted underneath the model fits. Estimates above 0 indicate looks to the target

The results of a similar model comparing language-matched children and children with ASD revealed a significant interaction between group and the cubic time term (*β* = −0.47, *SE* = 0.23, *p* = 0.04). As can be seen in Fig. 5, language-matched children and children with ASD looked away from the face at the beginning of the trial at a similar rate, but children with ASD returned to looking at the face before language-matched children. Thus, in general, children stayed looking at the face longer when there was noise in the background, perhaps an indication that they were using the face to gather information helpful for interpreting the speech signal. But children with ASD tended to look back toward the face sooner than did their language-matched counterparts.

### The impact of attention to the face on listening in noise

Finally, we examined whether those particular children who increased their looks to the face for noisy compared to quiet trials had an advantage in looks to the target on these noisy trials. This is, in essence, an examination of whether looking at the face is helpful for children. In the overall accuracy analysis, there was a strong correlation between how long children with ASD watched the face in the noise condition and their performance on the actual task; those who spent more time watching the face also spent a greater proportion of time looking at the correct (vs. incorrect) object, *r* = 0.626, *p* < 0.01. This was not the case for either their age-matched peers (*r* = 0.081) or for either group in quiet (children with ASD, *r* = 0.129; age-matched peers, *r* = 0.190). Perhaps the task was sufficiently easy in quiet, and for those without ASD, that they did not need the additional information provided by the face. This suggests the relationship between face-looking and accuracy is specific to those situations where it is most important, rather than simply being the result of differences among children in general task performance.

We completed the same analyses comparing the children with ASD with their language-matched controls. In general, the pattern of results was quite similar (see Table 3), with one exception: the correlation between time spent looking at the face in the noise condition and task performance, while still significant only for children with ASD, was somewhat stronger for their language-matched peers than it had been for the age-matched peers (in noise: *r* = 0.195; in quiet, *r* = 0.359). This trend toward a stronger correlation in these younger children may again suggest that the use of face information matters more in a difficult language-listening situation than in a simpler one.

**Table 3:** Proportion looking to the correct object (accuracy), proportion looking to the face, and correlation data of children with ASD and their peers in each of the four conditions

	Comparison with age-matched controls				Comparison with language age-matched controls		
	Children with ASD	CA controls	Statistical comparison		Children with ASD	LA controls	Statistical comparison
Proportion accuracy, NoFace-Quiet	66.3%	68.1%	*t* (16) = 0.38, *p* = 0.71		66.7%	70.2%	*t* (12) = 0.77, *p* = 0.46
Proportion accuracy, NoFace-Noise	62.8%	64.9%	*t* (16) = 0.64, *p* = 0.53		62.3%	64.7%	*t* (12) = 0.64, *p* = 0.54
Proportion accuracy, Face-Quiet	58.6%	68.9%	*t* (16) = 1.86, *p* = 0.08		58.1%	68.1%	*t* (12) = 1.54, *p* = 0.15
Proportion accuracy, Face-Noise	61.1%	67.4%	*t* (16) = 1.24, *p* = 0.23		64.3%	65.3%	*t* (12) = 0.19, *p* = 0.85
Looking to face, quiet	18.83 s	19.15 s	*t* (16) = 0.12, *p* = 0.90		19.45 s	19.71 s	*t* (12) = 0.05, *p* = 0.96
Looking to face, noise	19.34 s	18.53 s	*t* (12) = 0.38, *p* = 0.71		20.61 s	18.37 s	*t* (12) = 0.92, p = 0.37
Correlation, face looking and accuracy in quiet	0.13	0.19			0.05	0.36	
Correlation, face looking and accuracy in noise	0.63*	0.08			0.59*	0.20	

Note that in NoFace conditions, there are only two objects on the screen, and chance is thus 50%; in the Face conditions, there are three places on the screen that children can look at, and thus the base rate is 33%

To examine whether this influenced the time course of looks, we first constructed a base model similar to the one created to study *attention to the face*; however, in this model, the group was not included as a fixed effect. We used this base model to extract participant random effect estimates so that these values reflect effect sizes relative to the overall mean as opposed to effect sizes relative to each group’s mean. For each child, the random effect estimates from trials not in noise was subtracted from the estimate for trials in noise to obtain that child’s individual noise effect size. For ease of interpretation, a larger noise effect corresponds to a larger increase in looking to the face during noisy trials compared to quiet trials, whereas a smaller noise effect corresponds to a smaller increase in looking to the face during noisy trials compared to quiet trials. The final model examined how children’s noise effect size modulated target looks in noise and included noise effect as a fixed effect, restricted to trials presented with noise and where a face was present. Participant random effects were included on linear and quadratic polynomial time terms. Only effects and interactions with the noise effect are interpreted.

**Fig. 6:**
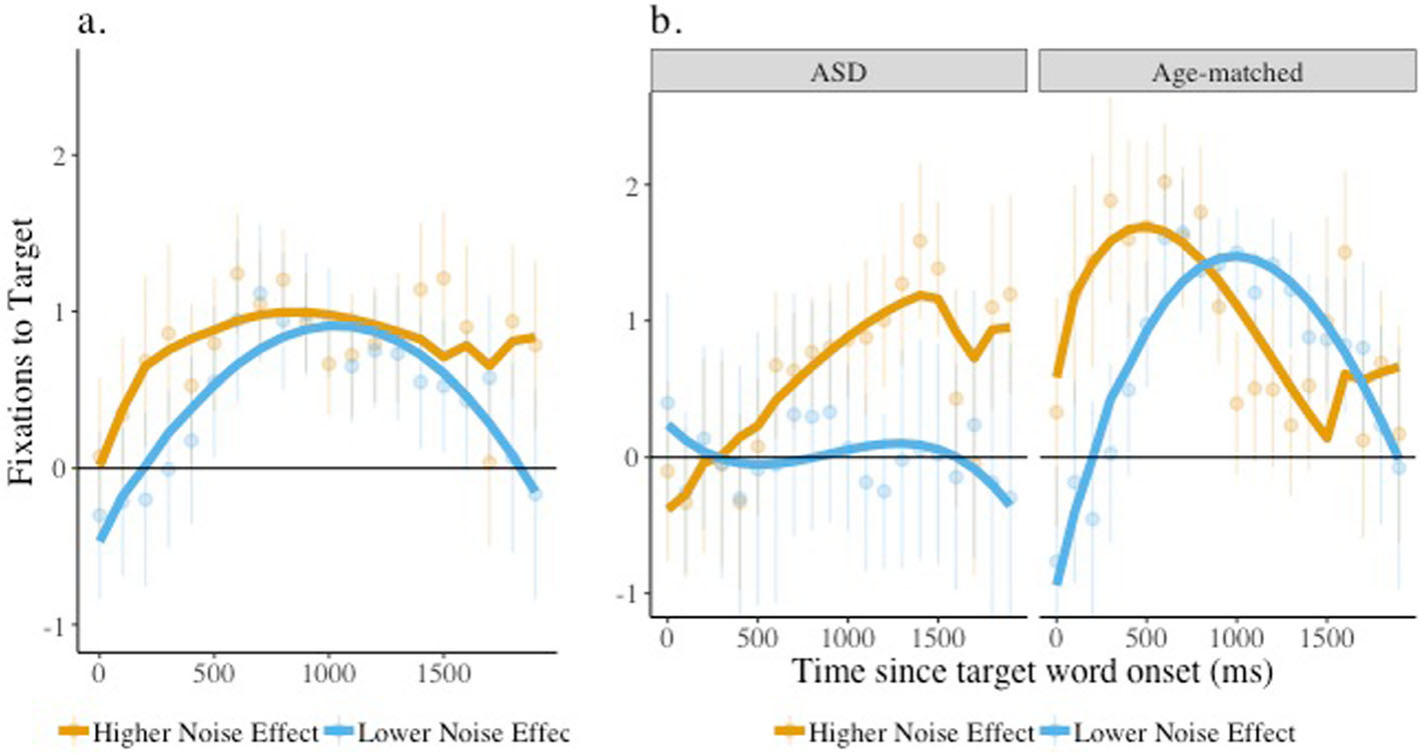
Target looks, noise effect, and noise effect by group interaction. Target image fixation is plotted comparing children with a high and low noise effect and children with ASD and age-matched peers (group) listening in noise when the face was present. The solid lines indicate the model fits for the significant effects including the noise effect (**a**) and the interaction between the noise effect and group (**b**). Raw means and standard errors are plotted underneath the model fits. Estimates above 0 indicate looks to the target.

The results of this model (Appendix 4) revealed a significant interaction between the noise effect and the linear time term (*β* = −7.38, SE = 2.70, *p* < 0.001). As can be seen in Fig. 6a, children who had a larger noise effect had a greater increase in target looks than children with a lower noise effect. The interaction between group, noise effect, and the linear time term was also significant (*β* = 12.21, SE = 3.43, *p* < 0.001). As can be seen in Fig. 6b, children with ASD who had a larger noise effect (who increased their looking to the face in noise) had greater increases in and peak target looks compared to those with a smaller noise effect. For age-matched children, target looks were initially higher for children with a larger compared to smaller noise effect, but this rapidly decreased perhaps due to these children switching their attention to the face.

The results of a similar model comparing language-matched children and children with ASD revealed no significant effect of or interaction with the noise effect, but only a significant effect of group, which was already captured in the analysis of target looks when the face was present.

In summary, then, those children with ASD who increased their looks to the face to aid them in the presence of noise relative to trials without noise were better able to interpret speech presented in noise and look to the correct object. Children with ASD who were less likely to make use of the face in this way subsequently failed to look appropriately when speech was presented in noise.

### Analyses among children with ASD

Finally, we examined whether overall accuracy in the listening task correlated with either ADOS scores or communication outcomes for those children with an autism diagnosis. Performance on the task in general (based on trials in quiet without the face) correlated significantly with ADOS social affect (ADOS-SA, raw) scores (*r* (16) = −0.42, *p* < 0.05, directional test), such that those with lower (less impaired) ADOS scores showed better performance (see [5] for similar results with older children). However, there was no correlation with the ADOS restricted and repetitive behavior (ADOS-RRB) scores (*r* (16) = 0.14). Not surprisingly, performance on the lab task also correlated significantly with Mullen receptive language scores (*r* (16) = 0.49, *p* < 0.05), with those children showing more accurate looking also demonstrating higher Mullen scores—suggesting both tasks are capturing a similar aspect of language skills within this population. That said, Mullen’s receptive language scores did not correlate with ADOS social affect scores (*r* (16) = 0.21, *p* > 0.05), suggesting that while both social affect and language scores relate to lab performance, they are not themselves strongly related. Thus, children with ASD who had better language skills performed better in the current task, as did those with fewer of the social difficulties associated with autism. However, because we have no measure of nonverbal IQ, we cannot differentiate whether the correlation with the ADOS is truly the result of social pragmatic differences or is driven by differences in nonverbal intelligence (see [41]).

## General discussion

Contrary to our initial hypotheses, children with ASD did not show particular difficulty listening to speech in the presence of background noise. Indeed, they performed well above chance at identifying the appropriate referent for a named object both in quiet and in noise. While noise did impact performance (at least when examining the time-course data), it did so in a manner similar for children with and without ASD. Yet despite a general similarity in performance across groups, the presence of noise did impact target looks to a greater extent in children with ASD than their language-matched peers specifically when a face was present. This difference is despite the fact that the level of noise in the current task (a signal-to-noise ratio of +5 dB SNR) was relatively slight, akin to that found during book-reading in toddler classrooms (a relatively quiet time in most classrooms; [69]; personal communication). Future work should explore performance in noise levels that more closely approximate typical home and school environments.

Prior work has reported that adults and adolescents with ASD have difficulty understanding speech in the presence of background noise, particularly when that noise consists of a single distractor voice [19, 24]. The present work does not support the notion of a specific impairment listening in noise; indeed, the effect of noise on young children with autism is quite mild, at least at this signal-to-noise level, which is encouraging. Rather, children with ASD appear less able to use additional cues to compensate for the impact of noise, such that noise has a greater functional impact on them than on their peers in situations where facial information is available. In that sense, the current results add nuance to prior findings about difficulties listening in noise among adolescents with ASD and suggests these may also impact young children who are still in the process of learning language.

But even though children with ASD might not be more affected by noise than their peers, this does not mean that noise is not a concern. Indeed, given the ubiquity of noise in educational settings, and the fact that children with ASD are already at risk for poorer language skills, any difficulty listening in noise is particularly worrisome, even if it is no greater than that faced by typical children. This is particularly the case if these children are less able to use other cues, such as the speaker’s face, to help compensate for the noise. Indeed, this might suggest that children with ASD would benefit from classroom modifications similar to those provided to children with hearing loss (another group that has difficulties listening in noise).

Any difficulty comprehending speech in noise, however, could be compensated for by looking at the speaker’s face—those children with ASD who attended more to the speaker’s face did not show the same impact of noise on their performance as did children who spent less time watching the face. One possibility is that children with relatively less impairment overall both look at the face more, and have better speech perception, perhaps because of better social abilities in general. Prior research suggests that time spent looking to non-face objects in a scene is related to social disability [70] and that social-communication abilities (such as joint attention) are strongly linked to vocabulary in ASD [71]. But it is also possible that watching the face may itself be a useful strategy for these children’s receptive language. Indeed, the benefit of the face was present only in the noise condition, suggesting it was used only in those situations where additional speech information was most needed.

Admittedly, caution needs to be taken in generalizing results from the current study to watching faces in natural settings—the current study only had three possible things to look at (the face and the two objects), whereas the real world provides an abundance of potential information sources. Moreover, the face in the current study was centered directly in front of the children, clearly visible, and at eye level. Our time-course analyses suggest that the ability to use facial cues while listening in noise impacted processing speed but failure to do so did not lead to a lack of recognition. However, prior research has demonstrated that neurotypical individuals regularly show benefits from having the face available [37, 72]. If watching a face is likewise a useful strategy for children with ASD, it could be clinically important, since face watching is likely to be a trainable skill for at least some individuals. Prior work has suggested that children with ASD can benefit from some types of intervention (e.g., specific auditory training, in [73]and lip-reading training in [74]). Given this, our findings suggest that future work should explore the efficacy of a training-based intervention geared toward encouraging children with ASD to look at the face of a speaker, particularly in noisy environments.

Previous studies have found that when instructed to watch a face talking, the children with ASD did so—they simply did not do so in situations where such information could be helpful but was not necessary [75]. Similarly, Massaro and Bosseler [74] report that children with ASD were less accurate at speechreading prior to specific training, and several studies report that adolescents with ASD show poorer audio-visual integration than their peers [19, 45]. Irwin and Brancazio [75]concluded that children with ASD failed to spontaneously use face information, but that training them to do so might be a useful intervention (see also [74]). The fact that those children in the present study who did spend more time watching the face of the speaker showed better performance supports this notion. Unlike Irwin and Brancazio’s study, our method of coding did not allow us to distinguish where on the face children were looking (i.e., whether children were looking at the speaker’s eyes or mouth), so we cannot determine whether children were lip reading to some degree or not. However, prior work suggests individuals with autism are more likely to watch the mouth of a person than his/her eyes even when that individual is not speaking [41]. We expect that visual speech information from the mouth in particular is likely to be one factor that helped children identify the correct word, and we expect that this effect would generalize outside of the laboratory setting (although further research is needed to determine whether this is in fact the case).

We also found that children with ASD who showed better overall language skills also performed better in this object identification task. This is rather unsurprising, as a fundamental assumption of this methodology is that eye-gaze movements reflect underlying language skills. However, we employed very simple sentences, with words chosen to be extremely well-known, such that we might have expected all children to find the task quite easy. Moreover, children with ASD often have difficulty with language assessments that require overt, deliberate responses, such as the Mullen Scales of Early Learning; the high correlation between test scores and eye gaze suggests that poor performance on the Mullen assessment may not be merely the result of behavioral difficulties/noncompliance. Of note, of the three children in the ASD group who had extremely low language scores on the Mullen Scales, one consistently looked longer to the named object across trials (71%), one showed a more moderate preference for the named object (57%), and one showed chance performance (48%). Perhaps gaze-based tasks would be a useful approach to differentiate language skills among children with low performance on explicit measures (see also [8]).

Children with ASD who showed more difficulties with social and affective skills, as indicated on the ADOS social affect scale, also performed more poorly in the current task. This suggests that noise may pose more of a problem for children with lower abilities; this is another topic for future research. However, other aspects of autism, such as those captured by the ADOS restricted and repetitive behaviors did not seem to relate to these language measures. This not only supports the notion that these are separate dimensions of autism spectrum disorders, but is suggestive that vocabulary acquisition (and thus receptive language skill) is specifically tied to social interactions, and not necessarily to more global measures of impairment.

Although a growing literature suggests that individuals with ASD have difficulty processing speech, particularly in the context of noise, the mechanisms responsible for these differences remain unknown, and the current results suggest these differences may be quite mild. Some work has suggested that individuals with ASD show physiological differences in a number of regions responsible for auditory processing (see [14] for a recent review), and may have less efficient neural encoding of basic acoustic information [14, 19] such as pitch [76]. These results seem to imply differences in fundamental auditory processing, even for a single stream of sound, which could become exacerbated as task demands increase. Research by Russo et al. [26] suggests that children with ASD show poorer auditory cortical responses to speech stimuli, even in quiet settings, and do not show any reduction in performance when noise is added to the environment (see also [25]), implying that children with ASD treat even speech in quiet akin to how their peers process speech in noise^5^. In contrast, we did find reduced speech perception performance in noise for children with ASD compared to language-matched controls (when the face was present); however, all of our participants were substantially younger than those tested by Russo et al., and speech perception may be a more demanding task at this young age. Moreover, our task required comprehension, whereas Russo et al. were measuring latency of cortical responses to a speech syllable. Thus, while basic auditory processing differences may contribute, it remains unclear what might be the underlying cause of children with ASD’s differences in processing speech in noise.

### Limitations

A limitation of our current study is the somewhat small participant size. This was due to our requirement that participants have research-reliable ADOS scores; most of our potential participants were already receiving clinical services, and thus had previously undergone ADOS testing, but which was often not research reliable. Many families of children with autism were reluctant to have their child sit through another ADOS testing session. This would likely have been less of an issue had we had a mechanism for reaching families with younger children. Relatedly, the fact that most children were already receiving clinical services may have impacted their performance.

Our decision to include children with low language skills is both a strength (in that such children are frequently excluded from language research) and a limitation (in that we were unable to find language matches for these children who were sufficiently mature to participate in the task). Another limitation is that testing in a laboratory session, with a face on a screen, is quite unlike real-world conversations with moving, 3-dimensional talkers. Nonetheless, our findings suggest that children with ASD are not as hampered by background noise as might have been predicted on the basis of the prior literature. Future work could examine this in more ecologically valid testing situations.

Finally, our study is limited in that our method of eye-tracking could not differentiate looks to the speaker’s mouth vs. elsewhere on the speaker’s face (such as her eyes). As such, we are unable to determine the extent to which children were using cues from the speakers’ mouth to help them understand speech in noise; this is another direction for future research.

## Conclusions

Young children with ASD, like their neurotypical peers, show poorer performance comprehending speech in the presence of another talker than in quiet. Moreover, children with more severe social and affective symptoms of ASD showed poorer comprehension of speech in general. However, those children with ASD who spent more time attending to the face of the target speaker appeared less disadvantaged by the presence of the distractor, indicating a potential path for future interventions.

## Data Availability

The coded data analyzed during the current study are available from the corresponding author on reasonable request. We are not able to provide raw video data as such would violate participant confidentiality.

## Appendix 1

### Output of the mixed effects noise model for target fixations

**Table 4:** Model output for the comparison between chronological age-match and ASD children

Fixed effects	Estimate	SE	df	*t* value	*p* value	Sig.
(Intercept)	0.61	0.09	67.93	7.08	< 0.001	****
Linear	0.78	0.38	63.25	2.09	0.04	**
Quadratic	−1	0.27	67.43	−3.72	< 0.001	****
Cubic	0.02	0.14	7793.05	0.16	0.88	
Condition	−0.23	0.11	35.26	−2.03	0.05	**
Group	−0.07	0.12	67.42	−0.55	0.58	
Linear: Condition	0.02	0.44	39.9	0.04	0.97	
Quadratic: Condition	0.34	0.37	57.04	0.92	0.36	
Cubic: Condition	−0.13	0.2	7792.47	−0.66	0.51	
Linear: Group	−0.66	0.53	62.76	−1.25	0.22	
Quadratic: Group	0.33	0.38	66.75	0.88	0.38	
Cubic: Group	0.08	0.2	7793.85	0.4	0.69	
Condition: Group	0.02	0.16	35.13	0.15	0.88	
Linear: Condition: Group	0.52	0.63	39.71	0.83	0.41	
Quadratic: Condition: Group	−0.37	0.52	56.78	−0.71	0.48	
Cubic: Condition: Group	−0.23	0.28	7793.26	−0.83	0.41	

Model construction: logitAdjusted ~ (Linear + Quadratic + Cubic)×Condition×Group + (1 + Linear+Quadratic|subject) + (1 + Linear+Quadratic|subject: Condition)

Significance codes: *p* < 0.001 = ****; < 0.01 = ***; < 0.05 = **; < 0.1 = *

**Table 5:** Model output for the comparison between language-matched and ASD children

Fixed effects	Estimate	SE	df	*t* value	*p* value	Sig.
(Intercept)	0.46	0.1	51.83	4.82	< 0.001	****
Linear	0.61	0.42	49.86	1.43	0.16	
Quadratic	−1.36	0.2	51.44	−6.71	< 0.001	****
Cubic	0.18	0.16	6003.7	1.14	0.26	
Condition	0.05	0.13	41.29	0.35	0.73	
Group	0.12	0.14	51.9	0.87	0.39	
Linear: Condition	0	0.54	28.08	0.01	0.99	
Quadratic: Condition	0.47	0.29	51.17	1.65	0.1	
Cubic: Condition	−0.58	0.23	6003.19	−2.53	0.01	**
Linear: Group	−0.35	0.6	49.98	−0.58	0.56	
Quadratic: Group	0.29	0.29	51.93	0.99	0.32	
Cubic: Group	−0.22	0.23	6005.85	−0.96	0.34	
Condition: Group	−0.31	0.19	41.29	−1.64	0.11	
Linear: Condition: Group	−0.04	0.76	28.08	−0.05	0.96	
Quadratic: Condition: Group	0.14	0.41	51.26	0.34	0.73	
Cubic: Condition: Group	0.34	0.32	6004.69	1.06	0.29	

Model construction: LogitAdjusted ~ (Linear + Quadratic + Cubic)×Condition×Group + (1 + Linear+Quadratic|subject) + (1 + Linear+Quadratic|subject: Condition)

Significance codes: *p* < 0.001 = ****; < 0.01 = ***; < 0.05 = **; < 0.1 = *

## Appendix 2

### Output of the mixed effects face present model for target fixations

**Table 6:** Model output for the comparison between chronological age-match and ASD children on trials where the face was present

Fixed effects	Estimate	SE	df	*t* value	*p* value	Sig.
(Intercept)	0.44	0.12	58.67	3.55	< 0.001	****
Linear	0.25	0.47	54.81	0.53	0.6	
Quadratic	−0.68	0.37	58.27	−1.84	0.07	*
Cubic	0.46	0.19	4374.75	2.49	0.01	**
Condition	0.06	0.14	39.2	0.44	0.66	
Group	−0.08	0.17	58.32	−0.47	0.64	
Linear:Condition	−0.37	0.57	28.75	−0.65	0.52	
Quadratic: Condition	−0.41	0.5	52.95	−0.81	0.42	
Cubic: Condition	−0.43	0.27	4378.7	−1.57	0.12	
Linear: Group	−0.36	0.67	54.75	−0.54	0.59	
Quadratic: Group	−0.34	0.52	58.37	−0.65	0.52	
Cubic: Group	−0.52	0.26	4375.59	−1.98	0.05	**
Condition: Group	−0.17	0.19	39.68	−0.86	0.39	
Linear: Condition: Group	0.98	0.81	29.48	1.21	0.24	
Quadratic: Condition: Group	1.17	0.71	53.78	1.64	0.11	
Cubic: Condition: Group	0.23	0.39	4389.75	0.59	0.55	

Model Construction: LogitAdjusted ~ (Linear + Quadratic + Cubic)×Condition×Group + (1 + Linear+Quadratic | subject) + (1 + Linear+Quadratic | subject:Condition)

Significance codes: *p* < 0.001 = ****; < 0.01 = ***; < 0.05 = **; < 0.1 = *

**Table 7:** Model output for the comparison between language-matched and ASD children on trials where the face was present

Fixed effects	Estimate	SE	df	*t* value	*p* value	Sig.
(Intercept)	0.59	0.13	45.02	4.59	< 0.001	****
Linear	0.38	0.51	46.75	0.74	0.46	
Quadratic	−0.02	0.38	44.2	−0.06	0.96	
Cubic	0.54	0.22	3262.23	2.47	0.01	**
Condition	0.07	0.14	25.45	0.52	0.61	
Group	−0.23	0.18	44.81	−1.26	0.21	
Linear: Condition	0.24	0.62	22.09	0.39	0.7	
Quadratic: Condition	−0.77	0.5	37.65	−1.52	0.14	
Cubic: Condition	−0.34	0.3	3277.52	−1.1	0.27	
Linear: Group	−0.89	0.72	46.56	−1.23	0.22	
Quadratic: Group	−1.26	0.54	43.24	−2.33	0.02	**
Cubic: Group	−0.69	0.31	3295.83	−2.24	0.03	**
Condition: Group	−0.05	0.2	26.61	−0.26	0.79	
Linear: Condition: Group	1.05	0.89	23.89	1.18	0.25	
Quadratic: Condition: Group	1.83	0.73	39.71	2.53	0.02	**
Cubic: Condition: Group	0.23	0.44	3291.63	0.52	0.6	

Model Construction: LogitAdjusted ~ (Linear + Quadratic + Cubic)×Condition×Group + (1 + Linear+Quadratic|subject) + (1 + Linear+Quadratic|subject: Condition)

Significance codes: *p* < 0.001 = ****; < 0.01 = ***; < 0.05 = **; < 0.1 = *

## Appendix 3

### Output of the mixed effects face preference model for face fixations

**Table 8:** Model output for the comparison between chronological age-match and ASD children on trials where the face was present

Fixed effects	Estimate	SE	df	*t* value	*p* value	Sig.
(Intercept)	−0.16	0.14	48.06	−1.19	0.24	
Linear	0.63	0.29	68	2.16	0.03	**
Quadratic	1.06	0.24	60.97	4.35	< 0.001	****
Cubic	−0.25	0.14	7953.99	−1.83	0.07	*
Condition	0.14	0.11	34.13	1.21	0.24	
Group	0.02	0.19	48.06	0.1	0.92	
Linear: Condition	−0.75	0.41	67.99	−1.82	0.07	*
Quadratic: Condition	0.24	0.28	35.42	0.86	0.4	
Cubic: Condition	0.73	0.2	7953.99	3.74	< 0.001	****
Linear: Group	−0.38	0.41	68	−0.92	0.36	
Quadratic: Group	0.04	0.34	60.97	0.1	0.92	
Cubic: Group	−0.01	0.2	7953.99	−0.07	0.94	
Condition: Group	0.12	0.16	34.13	0.75	0.46	
Linear: Condition: Group	0.84	0.59	68.02	1.44	0.16	
Quadratic: Condition: Group	−0.25	0.39	35.46	−0.63	0.53	
Cubic: Condition: Group	−0.04	0.28	7954.56	−0.13	0.9	

Model Construction: LogitAdjusted ~ (Linear + Quadratic + Cubic)×Condition×Group + (1 + Linear+Quadratic|subject) + (1 + Linear+Quadratic|subject: Condition)

Significance codes: *p* < 0.001 = ****; < 0.01 = ***; < 0.05 = **; < 0.1 = *

**Table 9:** Model output for the comparison between language-matched and ASD children on trials where the face was present

Fixed effects	Estimate	SE	df	*t* value	*p* value	Sig.
(Intercept)	−0.07	0.14	42.58	−0.52	0.61	
Linear	−0.09	0.42	47.47	−0.22	0.82	
Quadratic	1	0.26	49.88	3.84	< 0.001	****
Cubic	0.23	0.16	6084	1.45	0.15	
Condition	−0.07	0.14	27.73	−0.51	0.62	
Group	0	0.19	42.58	−0.01	0.99	
Linear:Condition	−0.12	0.49	37.35	−0.25	0.8	
Quadratic:Condition	−0.62	0.33	34.38	−1.9	0.07	*
Cubic:Condition	0.37	0.23	6084	1.64	0.1	
Linear:Group	0.4	0.59	47.47	0.67	0.51	
Quadratic:Group	0.22	0.37	49.88	0.59	0.56	
Cubic:Group	−0.47	0.23	6084	−2.05	0.04	**
Condition:Group	0.36	0.2	27.73	1.83	0.08	*
Linear:Condition:Group	−0.01	0.7	37.35	−0.01	0.99	
Quadratic:Condition:Group	0.69	0.47	34.38	1.48	0.15	
Cubic:Condition:Group	0.29	0.32	6084	0.9	0.37	

Model Construction: LogitAdjusted ~ (Linear + Quadratic + Cubic)×Condition×Group + (1 + Linear+Quadratic|subject) + (1 + Linear+Quadratic | subject:Condition)

Significance codes: *p* < 0.001 = ****; < 0.01 = ***; < 0.05 = **; < 0.1 = *

## Appendix 4

### Output of the mixed effects face in noise model for target fixations

**Table 10:** Model output for the comparison between chronological age-match and ASD children on noisy trials where the face was present

Fixed effects	Estimate	SE	df	*t* value	*p* value	Sig.
(Intercept)	0.81	0.23	33.9	3.54	< 0.001	***
Linear	−1.09	0.94	32.83	−1.16	0.26	
Quadratic	−1.99	0.77	34.29	−2.59	0.01	**
Cubic	0.61	0.28	566.7	2.18	0.03	**
Group	−0.42	0.32	33.9	−1.29	0.21	
Noise effect	−0.1	0.65	34.27	−0.15	0.88	
Linear: Group	2.22	1.33	32.88	1.68	0.1	
Quadratic: Group	1.6	1.08	34.11	1.48	0.15	
Cubic: Group	−0.9	0.39	570.23	−2.28	0.02	**
Linear: Noise effect	−7.38	2.7	33.61	−2.73	0.01	***
Quadratic: Noise effect	2.56	2.2	35.03	1.16	0.25	
Cubic: Noise effect	1.09	0.81	572.85	1.35	0.18	
Noise effect: Group	1.02	0.82	34.97	1.25	0.22	
Linear: Noise effect: Group	12.21	3.43	35.22	3.56	< 0.001	***
Quadratic: Noise effect: Group	−3.46	2.8	36.83	−1.23	0.22	
Cubic: Noise effect: Group	−0.7	1.06	585.4	−0.66	0.51	

Model Construction: LogitAdjusted ~ (Linear + Quadratic + Cubic)×Face Effect×Group + (1 + Linear+Quadratic|subject)

Significance codes: *p* < 0.001 = ****; < 0.01 = ***; < 0.05 = **; < 0.1 = *

**Table 11:** Model output for the comparison between Language-matched and ASD children on noisy trials where the face was present

Fixed effects	Estimate	SE	df	*t* value	*p* value	Sig.
(Intercept)	1.33	0.27	26.7	4.94	< 0.001	****
Linear	1	1	26.48	1.01	0.32	
Quadratic	−1	0.9	26.31	−1.11	0.28	
Cubic	0.4	0.29	429.82	1.36	0.18	
Group	−0.83	0.37	26.34	−2.21	0.04	**
Noise effect	0.47	0.64	26.51	0.73	0.47	
Linear: Group	−0.23	1.38	26	−0.17	0.87	
Quadratic: Group	0.7	1.24	26.02	0.57	0.58	
Cubic: Group	−0.49	0.41	432.36	−1.2	0.23	
Linear: Noise effect	−0.16	2.36	26.14	−0.07	0.95	
Quadratic: Noise effect	1.8	2.13	26.08	0.84	0.41	
Cubic: Noise effect	−0.84	0.69	428.16	−1.22	0.22	
Noise effect: Group	0.57	0.81	26.96	0.7	0.49	
Linear: Noise effect: Group	5.41	3.01	27.81	1.8	0.08	*
Quadratic: Noise effect: Group	−3.45	2.71	27.73	−1.27	0.21	
Cubic: Noise effect: Group	0.64	0.93	448.57	0.69	0.49	

Model Construction: LogitAdjusted ~ (Linear + Quadratic + Cubic)×Face Effect×Group + (1 + Linear+Quadratic | subject)

Significance codes: *p* < 0.001 = ****; < 0.01 = ***; < 0.05 = **; < 0.1 = *

## Footnotes

1 Some studies suggest that atypical gaze patterns increase with age [41], implying that children might be less affected than adults. However, these age-related differences in face processing may already be occurring by 3 or 4 years of age [41, 43] well within the age range of children tested here.

2 This is the only participant who was neither functionally monolingual nor strongly English dominant. We hesitated to remove this participant, given our small participant size; instead, we opted to include, but to do all analyses both with and without this participant; removing this child did not change the pattern of results in any way.

3 Because book-reading time is presumably among the most quiet periods in a toddler classroom.

4 It is worth noting that being blind to *condition* is not the same as being blind to *group*. Coders were not informed as to which children were in which group, but some children with ASD may have shown patterns of behavior that essentially unblinded the coders to their group membership. Yet coders remained blind to which condition occurred on each trial—they could not hear the stimuli and thus were not aware whether noise was present, nor whether the correct answer was to the left or right.

5 It is worth noting, however, that to encourage compliance, the participants watched (and listened to) a movie while testing was taking place; thus, even the “quiet” condition included some auditory background sounds.
